# Seroprevalence of Enterovirus A71 and Coxsackievirus A16 in Healthy People in Shandong Province, China

**DOI:** 10.1371/journal.pone.0162373

**Published:** 2016-09-09

**Authors:** Jian-xing Wang, Shuang-li Zhu, Jing Wang, Yi Lin, Yao-wen Pei, Da-peng Sun, Yong Zhang, Xian-jun Wang, Wen-bo Xu, Shu-jun Ding

**Affiliations:** 1 Institute for Viral Disease Control and Prevention, Shandong Provincial Key Laboratory of Communicable Disease Controland Prevention, Shandong Center for Disease Control and Prevention, Jinan Shandong 250014, China; 2 WHO WPRO Regional Polio Reference Laboratory and Key Laboratory of Medical Virology and Viral Diseases, National Health and Family Planning Commission of China, National Institute for Viral Disease Control and Prevention, Chinese Center for Disease Control and Prevention, Beijing 102206, China; 3 School of Public Health, Shandong University, Jinan, Shandong 250012, China; Duke-National University of Singapore Graduate Medical School, SINGAPORE

## Abstract

**Background:**

Hand, foot, and mouth disease has become very common in mainland of China in recent years, and enterovirus A71 and coxsackievirus A16 are its major etiologic factors. Here we investigated the seroprevalence of enterovirus A71 and coxsackievirus A16 based on a large group of healthy individuals in Shandong province, China.

**Methods:**

A total of 1378 healthy individuals were tested for serum neutralizing antibodies against enterovirus A71 and coxsackievirus A16 using a micro neutralization test.

**Results:**

The overall seroprevalence of enterovirus A71 neutralizing antibodies was 74.75%. It increased significantly from 48.84% in children aged 0–1 years old to 88.64% in those aged 20–29 years (p < 0.01) and decreased to 85.71% in adults > 40 years old with a significant gender-specific difference (p < 0.01). The overall coxsackievirus A16 antibody prevalence was 71.77%. It increased significantly from 39.53% in children aged 0–1 years to 80.68% in those aged 10–19 years (p < 0.01) and decreased to 75.63% in adults >40 years without a gender-specific difference. Nearly 50% of the children <1 year were susceptible to enterovirus A71 infection versus 40% to coxsackievirus A16 infection. Sample collection time and place also played a role in the enterovirus A71 and coxsackievirus A16 positive rates. The overall rates in January were significantly lower than those in April and August (p < 0.01); enterovirus A71 positive rates in Jinan city (capital city of Shandong province) were lower than those in Jining city and Zibo city (p < 0.05); and oxsackievirus A16 positive rates in Jining city were significantly higher than those in Jinan city and Zibo city (p < 0.01).

**Conclusion:**

There were significant differences among age groups, locations, and time points in the seroprevalence rates of enterovirus A71 and coxsackievirus A16 neutralizing antibodies in healthy people in Shandong province.

## Introduction

Hand, foot, and mouth disease (HFMD) was first reported in New Zealand in 1957, and human enterovirus A71 (EV-A71) and coxsackievirus A16 (CV-A16), members of the Picornaviridae family that were first isolated in 1958 and 1969, respectively [1], are the two major causative agents of HFMD that rarely cause acute neurological symptoms [2–4]. HFMD is a common infectious disease in young children, particularly in those <5 years, that is characterized by brief febrile episodes and a characteristic skin rash with or without oral ulcers, and HFMD outbreaks have been reported in many parts of the world, especially Southeast Asia [5–8]. In mainland of China, owing to a large number of deaths during a short period, HFMD emerged as a serious public health problem since a large HFMD outbreak associated with subgenotype C4 of EV-A71, started in Shandong province, China, in 2007 [9], and then spread throughout the country; in 2008, HFMD was classified as a category C notifiable infectious disease by the Ministry of Health in China. HFMD outbreaks are mainly caused by CV-A16 and/or EV-A71 infections [10–12]. The co-circulation of the two pathogens has been described previously [13–15]. Compared to CV-A16 symptoms, those caused by EV-A71 infection are more severe and lead to more serious complications and even fatalities [4,16,17].

Although immunogenicity in the maternal serum has been examined in some reports [18], the pattern of immune responses against HFMD has not been well studied in mainland of China. In addition, only a few studies of EV-A71 infections have been conducted in Singapore [19–21], Germany [22], Russia [23], Brazil [24], Vietnam [25], Thailand [26], and Cambodia [27]. However, further studies are required with regard to the distribution of immunogenicity against CV-A16 and EV-A71 infectious. This retrospective survey performed in Shandong province, China, aimed to provide fundamental data for the establishment of an immunization program against infection with EV-A71 and CV-A16 and investigate the seroepidemiology of EV-A71 and CA-V16 infections. In this study, a total of 1378 individuals were tested for serum neutralizing antibodies (NtAb) against EV-A71 and CV-A16 using a microneutralization test. Serum samples were collected from healthy people in seven different geographical counties of three cities (Jinan, Jining, and Zibo) in Shandong province in January, April, and August 2010, respectively, with no history of HFMD disease before or during the sample collection.

## Methods

### Ethics Statement

This study involved no human participants or human experiments. Only serum samples collected from healthy children for public health purposes were used, and written informed consent for the use of their clinical samples was obtained from their parents. This study was approved by the Ethics Committee of Preventive Medicine of Shandong Center for Disease Control and Prevention (Shandong CDC), and the methods were performed in accordance with approved guidelines.

### Sample collection

A total of 1378 anonymized samples collected from 1378 healthy individuals (one per subject from 652 males and 756 females, divided into seven different age groups: 0–1, 2–3, 4–9, 10–19, 20–29, 30–39, and >40 years) were tested to detect NtAb against EV-A71 and CV-A16 using a microneutralization test. Serum samples were collected in three cities (Jinan, Jining and Zibo) in Shandong province in January, April, and August in 2010, respectively. Previous epidemiological studies have indicated that January is generally the low-point of transmission whereas August is generally the peak in northern China [28].

### Neutralization test

The neutralization test was performed using a micro technique on a human rhabdomyosarcoma cell line as previously described, with some modifications [20]. Serum samples were initially diluted to 1:8, inactivated at 56°C for 30 minutes, incubated overnight at 4°C, and then diluted from 1:8 to 1:2048. Twenty-five microliters of the virus with a tissue culture infective dose (TCID_50_) of 100 was mixed with 25 μL of the appropriate serum dilution and incubated at 37°C for 2 hours. Finally, the rhabdomyosarcoma cell suspension (1 × 10^5^ cells/mL) was added to the mixture. The plates were then placed in a CO_2_ incubator at 36°C for 7 days and the potential cytopathogenic effect was determined visually. Cell control, serum control, virus control, and virus back titration were established on each plate. If the back titration showed 32–320 TCID_50_/well, then the test was considered valid. The EV-A71 strain (evolutionary branch C4a; GenBank accession number: EU703812) used in this study was isolated from a patient with HFMD in Anhui province in 2008 [29], while the CV-A16 strain (evolutionary branch B1b; GenBank accession number: GQ429229) was isolated from a patient with HFMD in Shandong province in 2007 [30]. The lowest dilution at which a cytopathic effect was observed in >50% of wells was considered the antibody titer of the serum sample, and a titer >1:8 was considered the cutoff for a positive antibody response [31–33]. To analyze the immunity level, three NtAb titer ranges were defined: 1:8–1:32 (low), 1:64–1:256 (medium), and 1:512–1:2048 (high).

### Statistical analysis

An antibody titer ≥1:8 was considered positive. The chi-square test was used to compare the distribution of NtAb-positive rates of EV-A71 and CV-A16 in different groups categorized by age, gender, specimen collection month, and specimen collection location. We conducted logistic regression analyses to examine the correlations between one main exposure (age, gender, specimen collection month, and specimen collection location) and EV-A71 and CV-A16 seroprevalence with adjustment for the other exposures. Odds ratios (ORs) and corresponding confidence intervals (CIs) for each category, compared with the reference group, were calculated.

## Results

### Overall EV-A71 and CV-A16 seroprevalence

**Fig 1:**
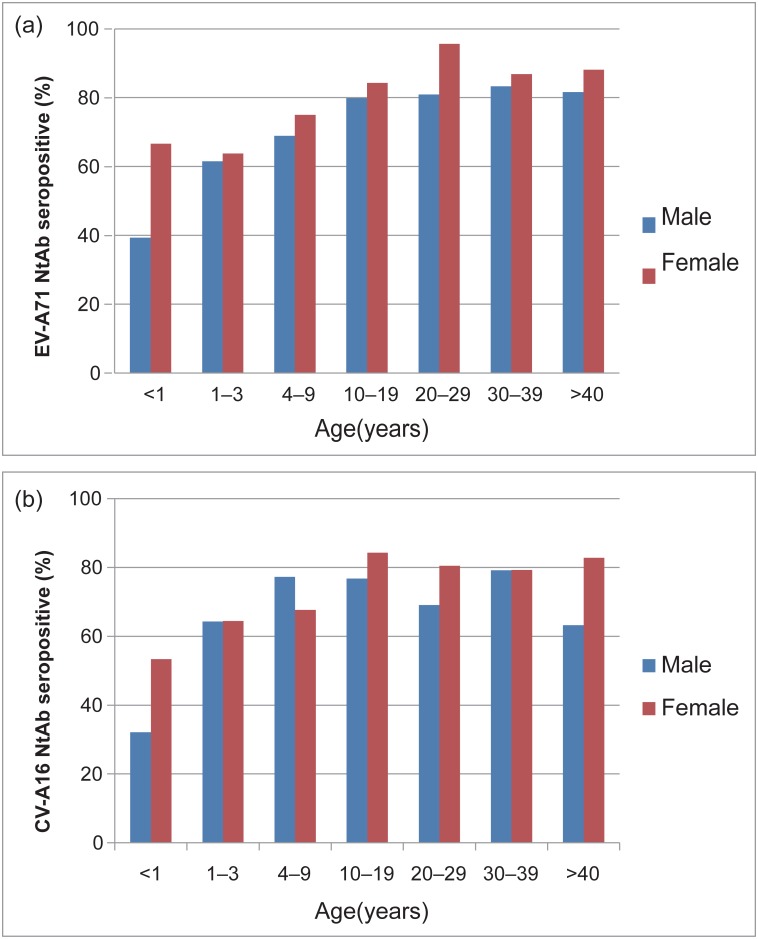
Age-rated and sex-dependent prevalence of neurtralizing antibodies to EV-A71 (upper panel) and CV-A16 (lower panel) in healthy individuals in Shandong province. (Male, blue bars; female, red bars).

EV-A71 and CV-A16 infections were widely present in the studied population, although all of the serum samples were collected from individuals who never suffered from HFMD. This result means that asymptomatic EV-A71 and CV-A16 infections are very common. Of the 1378 serum specimens tested in this study, the seropositive rates were 74.75% (1030/1378) for EV-A71 and 71.77% (989/1378) for CV-A16. Moreover, 45%–60% of the children aged <3 years had antibodies to EV-A71, which indicates that almost half of the children were susceptible to the infection (Fig 1 and Table 1).

**Table 1:** Seroprevalence of HEV71 and CAV16 in healthy people in Shandong province, China.

	EV-A71 Serostatus		P value	CV-A16 Serostatus		P value
	Negative	Positive		Negative	Positive	
Age, years (%)			<0.001			<0.001
<1	22 (51.16)	21 (48.84)		26 (60.47)	17 (39.53)	
1–3	123 (37.50)	205 (62.50)		117 (35.67)	211 (64.33)	
4–9	111 (27.96)	286 (72.04)		110 (27.71)	287 (72.29)	
10–19	37 (17.87)	170 (82.13)		40 (19.32)	167 (80.68)	
20–29	10 (11.36)	78 (88.64)		22 (25.00)	66 (75.00)	
30–39	11 (14.29)	66 (85.71)		16 (20.78)	61 (79.22)	
>40	34 (14.29)	204 (85.71)		58 (24.37)	180 (75.63)	
Overall	348 (25.25)	1030 (74.75)		389 (28.23)	989 (71.77)	
Sex, n(%)			<0.001			0.06
Male	194 (29.75)	458 (70.25)		200 (30.67)	452 (69.33)	
Female	154 (21.21)	572 (78.79)		189 (26.03)	537 (73.97)	

No significant difference in seropositive rate was observed between EV-A71 and CV-A16, indicating that the susceptibility to both viruses was the same in all age group. The EV-A71 seropositive rate was significantly higher in females (572/726; 78.79%) than in males (458/652; 70.25%) (p < 0.001), and the multivariable logistic regression analyses also supported this conclusion. However, no gender-specific difference was identified in CV-A16 seroprevalence (p > 0.05) (Tables 1 and 2). We compared the distribution of EV-A71 seroprevalence by age groups and found that it was 48.84% for those aged <1 year (n = 43), 62.5% for those aged 1–3 years (n = 205), 72.04% for those aged 4–9 years (n = 286), and >82% for all subjects aged >10 years. Compared with those aged <1 year, the seroprevalence was significantly increased in the older age groups, with an OR (95% CI) of 2.7 (1.4–5.2) for those aged 4–9 years, 4.8 (2.4–9.9) for those aged 10–19 years, 8.0 (3.2–19.7) for those aged 20–29 years, 6.1 (2.5–14.9) for those aged 30–39 years, and 6.1 (2.9–12.5) for those aged >40 years; there was no statistically significant difference, excepted for those aged 1–3 years, with an OR (95% CI) of 1.6 (0.8–3.0) (Table 2).

**Table 2:** Multivariable logistic regression of EV-A71 and CV-A16 seroprevalence.

	EV-A71	P value	CV-A16	P value
Variable	OR (95% CI)		OR (95% CI)	
Age in years				
<1	Reference		Reference	
1–3	1.6 (0.8–3.0)	0.17	2.6 (1.3–5.0)	0.005
4–9	2.7 (1.4–5.2)	0.003	4.1 (2.1–7.9)	<0.001
10–19	4.8 (2.4–9.9)	<0.001	6.5 (3.1–13.3)	<0.001
20–29	8.0 (3.2–19.7)	<0.001	4.6 (2.1–10.2)	<0.001
30–39	6.1 (2.5–14.9)	<0.001	5.9 (2.5–13.7)	<0.001
>40	6.1 (2.9–12.5)	<0.001	4.7 (2.3–9.5)	<0.001
Sex				
Male	0.7 (0.5–0.9)	0.01	0.9 (0.7–1.1)	0.22
Female	Reference		Reference	

Compared to that observed for EV-A71, the overall seroprevalence of CV-A16 was a little lower in the majority of age groups, but the difference was not statistically significant. There was a significant increase in the CV-A16 seroprevalence from 39.5% among infants <1 year of age to 64.3% for subjects 1–3 years old (p < 0.001). Compared with subjects <1 year of age, the seropositive rate increased in older age groups, with an OR (95% CI) of 2.6 (1.3–5.0) for those aged 1–3 years, 4.1 (2.1–7.9) for those aged 4–9 years, and 6.5 (3.1–13.3) for those aged 10–19 years and then decreased to 4.7 (2.3–9.5) for those >40 years of age. From the results, we can infer that infants <1 year of age were the susceptible population. The peak of the CV-A16 seroprevalence was observed in subjects 10–19 years old, with no further significant decline in the subsequent age groups.

### Titer distribution of NtAb in seropositive individuals and age-dependent immunity to EV-A71 and CV-A16 infections

**Fig 2:**
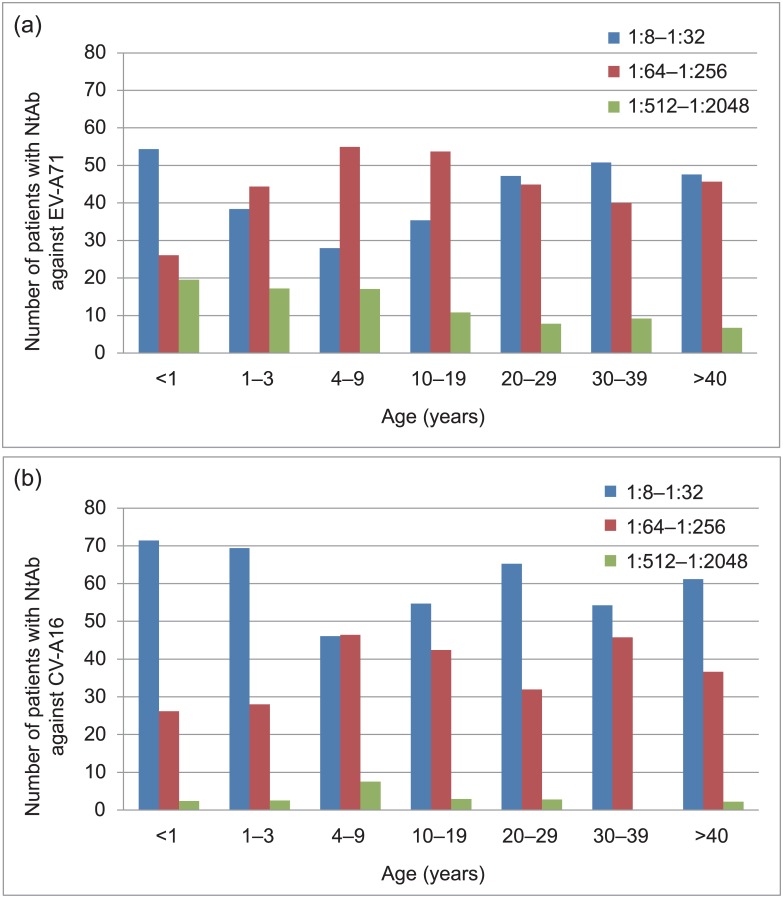
Titers of NtAb to EV-A71 (Panel A) and CV-A16 (Panel B). Only seropositive individuals of the study are included in the figure.

To analyze the immunity level, three NtAb titer ranges were defined: 1:8–1:32 (low), 1:64–1:256 (medium), and 1:512–1:2048 (high). Our analysis showed that the distribution of low and medium EV-A71 NtAb titers among the different age groups was inconsistent (Fig 2A). For the low NtAb titer, the percentages of three groups (aged 1–3, 4–9, and 10–19 levels) were <40%, while those of the remaining age groups were all >40% (Fig 2A). In contrast, for the medium NtAb titer, the lowest percentage (26%) was found in the first age group (< 1 year), while those of the remaining age groups were all >40% and the subjects 4–9 years had the highest percentage (approximately 55%) (Fig 2A). Unlike the trend observed with low and medium-level titers, high EV-A71 NtAb titers decreased as age increased.

In contrast, most people tested presented with low-level CV-A16 NtAb titers (Fig 2B), especially the <1, 1–3, and 20–29 year age groups. However, patients with medium-level NtAb titers (4–9, 10–19, 30–39 year age groups) comprised >40% of the study population, while those of the remaining groups comprised <40% of the study population, with patients aged <1 and 1–3 years accounting for <30% (Fig 2B). In particular, only a few people demonstrated high CV-A16 NtAb titers (Fig 2B).

### Influence of collection time points and geographic areas

We conducted a multivariable logistic regression according to specimen collection time and geographic areas to study their influence on the seropositive rate (Table 3). Our results showed that serum specimens collected in January had significantly lower seropositive rates of EV-A71 and CV-A16 NtAb than those of samples collected in April and August (both p < 0.01).

**Table 3:** Multivariable logistic regression of the geometric mean titer for EV-A71 and CV-A16 seropositive individuals.

Variable	EV-A71	P value	CV-A16	P value
	OR (95% CI)		OR (95% CI)	
Month of specimen collection				
January	0.6 (0.5–0.9)	0.004	0.5 (0.4–0.7)	<0.001
April	1.3 (1.0–1.9)	0.08	1.0 (0.7–1.4)	0.93
August	Reference		Reference	
Place of specimen collection				
Jinan	0.7 (0.5–1.0)	0.04	0.9 (0.7–1.2)	0.53
Jining	1.1 (0.8–1.5)	0.55	1.5 (1.1–2.0)	0.01
Zibo	Reference		Reference	

Meanwhile, the seroprevalence of EV-A71 in Jinan city was lower than those observed in Jining city and Zibo city (p < 0.05). The seroprevalence of CV-A16 in Jining city was higher than that observed in the other two cities (p < 0.05).

## Discussion

EV-A71 and CV-A16 are the major pathogens associated with sporadic cases and epidemics of HFMD, although 50–80% of infections are asymptomatic. In mainland of China, an outbreak of HFMD occurred in Shandong province in 2007 that was caused by a subgenotype C4 virus of EV-A71. A total of 1149 cases were reported; of them, 970 patients (84.4%) were <5 years old [9]. Nationwide HFMD outbreaks have occurred since 2008 [29], and the HFMD epidemic continues to infect Chinese children <5 years of age every year [28,34,35]. However, CV-A16 could co-circulate with EV-A71, causing very few severe illnesses [36]; EV-A71 seems to be more severe with significantly higher frequencies of serious complications and fatalities [17]. A serologic survey would be useful to understand the level of disease susceptibility and immunity against HFMD among different age groups of people. With NtAb detection, a guide for future immunization programs against HFMD could be developed. This report provides evidence of the improvement of disease monitoring and prediction measures against HFMD.

Our study revealed that the positive rates of NyAb against EV-A71 among children aged <3 years were significantly lower than those of people aged >10 years, while the positive rates of NtAb against CV-A16 among infants aged <1 year were significantly lower than those aged >10 years. With increasing age after 10 years, the positive rates of NtAb against EV-A71 and CV-A16 reached a plateau. Our data also indicate that most EV-A71 infections occur during childhood or early adolescence. Accordingly, the highest antibody titers were observed in these age groups. The low EV-A71 and CV-A16 seroprevalence in the young age groups indicates that infection before the (pre)school years is uncommon. This finding is also supported by the observation that a high proportion of children aged <5 years old were seronegative. The positive immune status for EV-A71 of children reached a steady state in children 4–9 years of age. Furthermore, the number of individuals with a high anti-EV-A71 NtAb level decreased as age increased. In contrast to EV-A71, a high anti-CV-A16 NtAb level was rare in all age groups, indicating that EV-A71 might account for the dominant cases in the HFMD epidemic process.

Immunity to EV-A71 and CV-A16 is mainly dependent on humoral factors, i.e. NtAb formation, although enteroviral infections also induce T-cell immunity [37]. It is currently unclear whether each individual with a low or undetectable NtAb level is susceptible to EV-A71 infection.

In mainland of China, C4a EV-A71 first led to a large-scale HFMD epidemic in Linyi city of Shandong province, where it was first confirmed that EV-A71 induced additional cases of HFMD-related deaths in mainland of China [9]. Previous studies suggested that the EV-A71 strain that caused large-scale HFMD outbreaks with a high number of severe cases and deaths in Fuyang city of Anhui province in 2008 and in Shangqiu city of Henan province in 2009 originated from Linyi city of Shandong province by molecular evolution [38]. Hence, studies on the HFMD epidemic and EV-A71 seroprevalence in Shandong province have a very important position in the history of the HFMD epidemic in mainland of China.

However, Shandong province is located in eastern China with a temperate monsoon climate. In contrast to tropical regions where infections occur at a high incidence throughout the year, HFMD outbreaks and epidemics in Shandong province primarily occur in summer and autumn [28,39]. Our study also found that the serum specimens collected in January (cold season) had significantly lower EV-A71 and CV-A16 NtAb seropositivity rates than those collected in April and August (warm season). These findings are in line with the HFMD epidemic and indicate that the enteroviral infection prevalence is seasonal. In addition to the factor of temperature, some other factors such as school holidays or the beginning of a school term may also be the causes of the formation of seasonal.

In Cambodia in 2012, a HFMD outbreak characterized by severe encephalitis with cardiovascular collapse and pulmonary edema seized international attention and resulted in the deaths of at least 54 children [27] and ultimately, the evolutionary branch C4a EV-A71 was identified as the cause, which is similar to the popular HFMD in mainland of China. Serum samples collected during 2000–2011 were screened for NtAb against EV-A71 and showed that the overall seroprevalence of EV-A71 NtAb was 93.1% (1:8 was set as the cutoff value), which showed a similar pattern in this study [27]. A number of studies demonstrated the high seroprevalence of EV-A71 NtAb among children several years before and after the large-scale HFMD outbreaks and indicated that the EV-A71 seropositivity rate increased with age but showed no significant sex-specific difference [25,26]. However, the seroprevalence of EV-A71 varies significantly among different regions of the world in countries without large-scale HFMD outbreaks, e.g. the reported EV-A71 seroprevalence was only 12% in subjects 1–4 years of age in Germany [22] and 20.2% in subjects 1–3 years of age in Brazil [40].

Studies have also suggested that geographical differences in EV-A71 and CV-A16 infections in different parts of China, particularly northeast China, where EV-A71 and CV-A16 infections were inactive mainly due to the cold climate, low population density, and other factors [33]. In the same province, the seroprevalence of EV-A71 or CV-A16 may be completely different in different geographic regions. Among the three cities in this study, in 2010, the seropositive rate of EV-A71 in Jinan city was the lowest, while the seropositive rate of CV-A16 in Jining city was the highest. There were no apparent links between EV-A71 seroprevalence and population density due to the three cities with similar population densities. However, the prevalence of EV-A71 and CV-A16 may change after enough susceptible persons were accumulated in the area with lower seropositive rate of the corresponding viruses. Therefore, public health measures to control the spread of EV-A71 and CV-A16 should be devised according to the different regional characteristics of areas in mainland of China [33].

Although EV-A71 (evolutionary branch C4a) [38] and CV-A16 (evolutionary branches B1a and B1b) [30] are widely believed to be the most prevalent serotypes in circulation in mainland of China, this situation may change under certain circumstances. Many other genotypes and subgenotypes of EV-A71 are currently circulating in neighboring countries and regions, and some sporadic importations of other genotypes and subgenotypes of EV-A71 have already been reported [41,42], however, they never became predominant genotypes in mainland of China up to now. Even if this happens, a cross neutralization study with nine EV-A71 strains belonging to nine genotypes and subgenotypes (A, B3, B4, B5, C1, C2, C3, C4, and C5) showed remarkable cross-neutralizing reactivity to these different genotype EV-A71 strains [43] which means C4 subgenotype EV-A71 strain could be used in the seroepidemiology study in mainland of China and can give an true neutralizing antibody levels against all genotypes of EV-A71. However, other serotypes such as CV-A6 were circulating in mainland of China and became the predominant enteroviruses that caused HFMD in 2013 and 2015 [36,44–47]. Inactivated EV-A71 vaccines that developed based on the C4 genotype strain circulating throughout mainland of China were licensed in the end of 2015 [48–50], and will be used to prevent EV-A71–related HFMD in 2016; although an inactivated EV-A71 vaccine derived from C4 subgenotype strain could be effectively used to induce adequate protective immunity against infection by most of the predominant circulating EV-A71 strains [43], it doesn't work for CV-A6. Thus, a seroprevalence study of other enteroviruses such as CV-A6 using the same serum samples is also very important and can help predict the efficacy of a mass vaccination program that specifically targeted EV-A71.

In conclusion, EV-A71 or CV-A16 infection is very common in Shandong province. Our results were generally consistent with those obtained from neighboring provinces, such as Guangdong, Shanghai, Jiangsu and Anhui [18,32,33,51,52]. This study reflects that herd immunity against EV-A71 infection is lower in young children in Shandong province even after the EV-A71 outbreak in 2007 [9]. Therefore, an immunologic protective barrier cannot be built through natural infection. The significant increases in the HFMD morbidity and mortality have caused an enormous public health burden. Unfortunately, there are no effective drugs against enteroviral infectious, and severe cases often progress very rapidly. Therefore, a safe and effective vaccine and a targeted effective therapeutic agent against EV-A71 to control EV-A71 outbreaks is urgent needed to reduce EV-A71-related disease burdens and fatalities.
